# Individual activity of forest rodents correlates to pathogen communities

**DOI:** 10.1038/s41598-026-51276-6

**Published:** 2026-05-09

**Authors:** Jana A. Eccard, Jasmin Firozpoor, Mario Escobar, Maxime Galan, Nathalie Charbonnel

**Affiliations:** 1 Animal Ecology, Institute for Biochemistry and Biology, University of Potsdam, Potsdam, Germany; 2 CBGP, INRAE, CIRAD, IRD, Institut Agro, Univ Montpellier, Montpellier, France

**Keywords:** *Bartonella*, *Borrelia*, *Mycoplasma*, *Sarcocystidae*, *Apodemus*, *Clethrionomys*, *Myodes*, Behaviour, Animal personality, Activity, Diseases, Ecology, Ecology, Microbiology, Zoology

## Abstract

Animal personality influences organismal interactions and individual habitat use. Rodents are zoonoses reservoirs and often exposed to several pathogens simultaneously, potentially resulting in interdependence of infections and susceptibility to infection. Still, entire pathogen communities are rarely investigated, even though, given rodents ubiquity in human settlements, understanding the link between animal personality and pathogenesis is an important public health issue. We investigated the association of animal personality with pathogen communities in wild rodents, analysing ectoparasite occurrence and pathogenic bacteria of 93 individuals belonging to 3 species from urban and forest areas around Potsdam, Germany. Individual personality was quantified using a combination of open-field and dark-light test. Rodents were then euthanised and screened for pathogens in the spleen through 16 S rRNA amplicon sequencing, and ectoparasites were collected. We detected 6 pathogenic bacteria and 3 ectoparasite taxa. Host species and sampling time explained most of the variation in pathogen associations, but within each genus, 7–9% of the variation was explained by animal personality. Active rodents were more likely infected by *Bartonella* than less active ones. Bold animals had lower tick infestation probabilities. Thus, animal personality contributes to the distribution and prevalence of pathogens in wild rodents, and should be considered in epidemiology and disease management.

**Supplementary Information:**

The online version contains supplementary material available at 10.1038/s41598-026-51276-6.

## Introduction

Zoonotic diseases are an emerging global threat^1^and understanding transmission patterns and pathogen occurence and prevalence within the animal population is critical. Variation in pathogen prevalence among individual animals may not only depend on the individual immunosystem, but also on a range of behavioural correlates that may affect space use and sociability of animals. For example, parasite load and parasite transmission can relate to behavioural traits (e.g^2,3^.,). More exploratory chipmunks (*Tamias minimus*) host a greater abundance of ectoparasites^4^. Bolder grey squirrels (*Sciurus carolinensis*) are more likely to be infected by gastro-intestinal helminths^5^. Less explorative multimammate mice were more likely to be infected by MORV virus^6^ in relation to their more exploratory conspecifics. Conversely, the presence of certain pathogens can influence animal behaviour. *Toxoplasma gondii*, for example, alters behaviour in mice making them bolder^7^; honeybees (*Apis mellifera*) alter their behavioural physiology after being infected by *Nosema ceranae*^8^, and exposure to the bacterium *Serratia marcescens* during development affects the expression of boldness in crickets^9^. These examples highlight the strong links and feedbacks between host behaviour and pathogen prevalence.

The concept of animal personality may capture some of the behavioural variation linked to pathogen prevalence. Animal personality is commonly defined as the between-individual differences in behaviour that persist through time and contexts^10,11^. Animal personality can also be linked to fitness and can be partly heritable^10^. Personality traits are particular aspects of an individual’s behavioural repertoire^12^, and out of these traits, five major categories including boldness, aggressiveness, activity, exploration, and sociability have been studied extensively^13,14^. However, not much is known about how animal personality is linked to the composition of pathogen communities, and to which single pathogens, even though this could crucially improve understanding disease transmissions and their spread in populations. Especially when considering that only a small proportion of animals is responsible for a large proportion of the transmission of a pathogen (“80/20 rule”^15^) due to the typical negative-binomial distribution of infection severity and parasiste loads, it might be crucial to understand which traits distinguish individuals that are highly infected. Personality traits such as sociability, exploration and boldness have been associated to this asymmetry of transmission and infection. These traits have been focused on independently showing their separate effects on diseases transmission, as well as considered together as suits of correlated behavioural traits^16^. When focusing on the single effect of exploration, an experiment on little brown bats (*Myotis lucifugus*) using a fluorescent powder as a substitute pathogen showed that the more explorative male bats were more likely to transmit and acquire infections^3^ than the less explorative males. In domestic cats boldness was positively linked to the infection probability of Feline Immunoficiency Virus, a lethal disease^17^. Furthermore, personality traits can alter where animals shed infectious agents in the environment^15^ as space use is often linked to animal personality^18^ and can shape whether individuals spatially interact with those of other species’^19^. Sociability may affect contact rates, which are important for microbiome diversity^20^ but may also affect pathogen exchange, direct disease transmission and infection patterns, as shown in three-spined sticklebacks^21^.

In deer mice (*Peromyscus maniculatus*), bolder individuals were more often in contact with conspecifics and thus also more likely to be infected by hantavirus^22^ and in Eastern grey squirrels bolder and more explorative individuals were more likely infected by gastro-intestinal helmiths^5^. These examples show that animal personality traits affect pathogen loads, encounter rates and patterns of disease transmissions and that not only effects of single personality traits are relevant but that suits of correlated behavioural traits, i.e. behavioural syndromes, play an important role as well, as several traits might act together in creating the observed infection patterns. Here we aim to study an entire pathogen community, and relate occurrence of the single pathogens as well as community richness and composition to animal personality in forest rodent communities.

Ectoparasites are known vectors for zoonotic infectious agents^23,24^. Links between animal personality and ectoparasite presence are equivocal: bolder ground squirrels carried more ticks^4^, but shyer bank voles tended to use higher vegetation cover and carried a higher tick load than bold conspecifics^25^. More explorative firebugs had higher loads of ectoparasitic mites^26^. Ectoparasite load alone can impact fitness and overall health^27,28^, linking them to pathogen transmission for their hosts and humans^29^. Animal personality may in part explain the likelihood of acquiring ectoparasites. In most small mammal species investigated so far, bolder or more exploratory individuals had larger home ranges^30^ or larger ranges but less overlap with conspecifics in these ranges^18,31^ than shyer or less explorative individuals. Considering that most ectoparasites are free-living, personality-mediated space use and microhabitat choice may contribute to higher ectoparasite loads through an increase in host-ectoparasite encounters. We therefore added ectoparasite infestations to the analysis of pathogen communities in forest rodents.

Rodents are the most abundant and diversified group among mammals, representing more than 40% of mammalian species^32^. They are reservoir hosts for multiple zoonotic diseases, of which to date 66 were identified^33^. Their worldwide distribution and proximity to human settlements make the risk of zoonotic spillovers to humans highly likely. Rodent ectoparasites have been identified as hosts for agents responsible for diseases such as ehrlichiosis, Lyme disease, tick-borne encephalitis, bubonic plague, and murine typhus among others^23,24^. To understand prevalence and pathogen dynamics in this group, the co-variation of pathogens and animal personalities may thus be important for disease prevention.

In this study we take a holistic approach trying to understand how host behaviour affects infection risk in natural zoonotic reservoirs. This not only would improve our theoretical understanding of disease dynamics but also would contribute to advance epidemiological surveillance and disease management. To achieve this, we applied molecular methods to detect multiple pathogens with no a priori knowledge, from free living rodents in parks and gardens. We also collected and counted ectoparasites, which can be involved in transmission of bacterial infectious agents. To add knowledge on behavioural drivers of pathogen occurrence, we collected individual-based, behavioural information with standard behavioural tests and repeated testing. We hence quantified consistent individual traits, that might collectively modulate infection risks across multiple pathogens simultaneously. We made use of an ongoing rodent collection a pan-european surveillance study investigating rodent pathogen diversity in parks and forests across Europe (BiodivERsA-BioRodDis, https://www6.inrae.fr/biodiversa-bioroddis) and added an additional, in-situ behavioural information layer for each individual, before the terminal collection of the rodent. We hypothesized that single pathogen infections and pathogen communities vary among animals with different behavioural phenotypes, and that the richness of pathogen communities may correlate to personality axes. We expect bolder and more active individuals of all species in the rodent community to have the higher infection probability for pathogens and ectoparasites, compared to shyer and less active individuals, due to their larger home ranges increasing the chance for infection, and higher contact rates with conspecifics.

## Results

### Behaviour

**Fig. 1:**
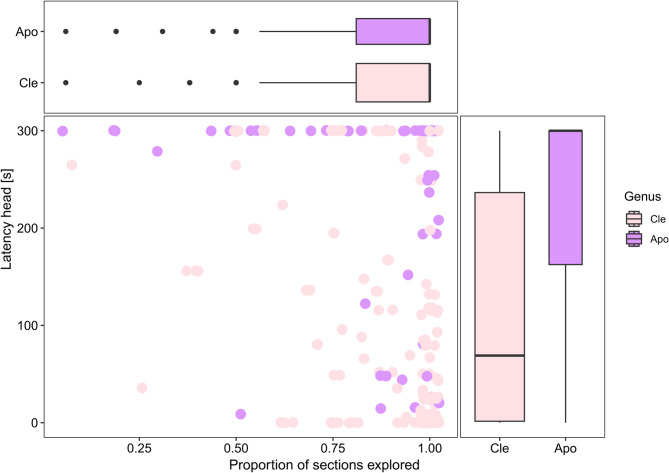
Behavioural variables chosen as quantitative measures for boldness (latency head) and activity (proportion of floor sections explored) in 93 wild forest rodents in their first test. Dots represent the values of individuals (scatterplot), boxplots and dot colours the species *Clethrionomys glareolus* (Cle, 41 individuals), and the genus *Apodemus *(Apo, 47 individuals).

In total 186 individuals, 79 *Clethrionomys glareolus*, 56 *Apodemus agrarius* and 51 *A. flavicollis*, were captured in a park and a forest near Potsdam, Germany, and their behaviour was quantified. Both activity and boldness differed between the two genera, with *Apodemus* being bolder (more likely to emerge from shelter) and tended to be more active (Table 1, A4, A5 and Fig. 1) than *Clethrionomys glareolus*. Behavioural variables did not differ between the two *Apodemus* species (activity: Chi2 = 0.528, df = 1, *p* = 0.468; boldness Chi2 = 0.01, df = 1, *p* = 0.918; Table A5).

**Table 1:** Behavioural variables from an emergence test (emergence from shelter in 5 min, representing boldness) and an open field test (proportion of floor sections covered in 5 min, representing activity) in 186 forest rodents analysed with generalized linear mixed models. Reference for season was autumn, for site was park, for genus was apodemus, for sex was female. Repeats were numbered (first to third test, however not all individuals were tested repeatedly). Individual ID was used as random factor.

	Emergence				Proportions sections			
	Estimate	SE	z value	Pr(>|z|)	Estimate	SE	z value	Pr(>|z|)
(Intercept)	**−11.41**	**2.77**	**−4.12**	**< 0.001**	**2.10**	**0.84**	**2.50**	**0.013**
Season (Spring)	0.05	1.91	0.03	0.978	−0.42	0.57	−0.74	0.460
Site (forest)	−0.90	1.82	−0.50	0.619	0.86	0.61	1.41	0.160
Genus (Cle)	**22.73**	**2.83**	**8.03**	**< 0.001**	−0.93	0.51	−1.81	0.070
Sex (males)	0.21	1.82	0.17	0.870	0.72	0.51	1.40	0.163
Repeat	0.22	1.82	0.12	0.907	0.21	0.49	0.43	0.664
Marginal R^2^	0.05				0.16			
Conditional R^2^	1.00				0.16			
AIC	149.08				126.75			

Shown are factor estimates of the fixed effects, the standard error (SE) of the fixed effects as well as the *t*-value. Additionally, the amount of variance explained by the fixed effects alone (marginal *R* squared) and the fixed and random effects together (conditional *R* squared) and the CI value are shown.

### Pathogen and ectoparasites

We detected pathogens in the spleen of 71 animals, and both spleen pathogens and ectoparasites in 86 out of 93 animals. In 22 animals no pathogens were detected, 83% of these were *C. glareolus*. Uninfected *C. glareolus* were found at both study sites while uninfected *A. agrarius* were only found at the park site, and uninfected *A. flavicollis* were only found at the forest site (Table 2). Pathogens correspond to 6 bacterial genera and one Apicomplexa family (Sarcocystidae). *Mycoplasma haemomuris* (mych), *Bartonella* (bart), and *Candidatus Neoehrlichia mikurensis* (neom), were commonly detected in both rodent taxa, while *Mycoplasma coccoides (mycc)* was detected only in the genus *Apodemus*, while Sarcosystidae (sarc) was found mainly in *C. glareolus. Francisella Orientia*, and *Anaplasma* were negative in all tested individuals. 

**Table 2:**
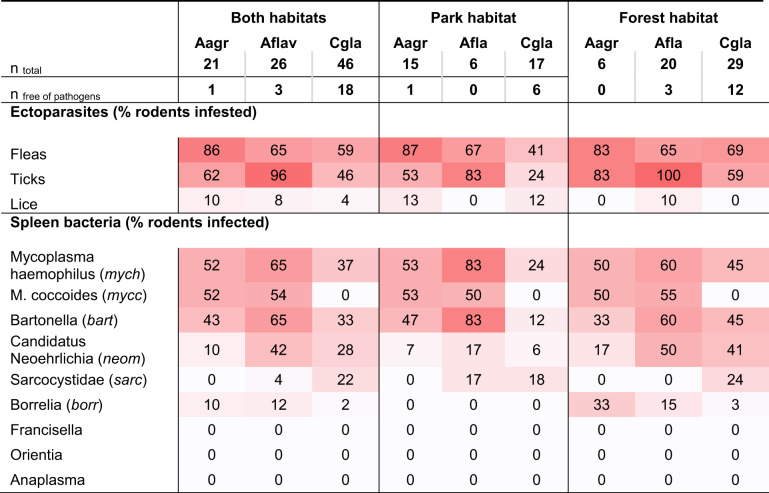
Ectoparasite and pathogen occurrence in percent of samples from three species of forest rodents (Aagr: *Apodemus agrarius*; Afla: *Apodemus flavicollis*; Cgla: *Clethrionomys glareolus*) in Germany sampled in autumn 2021 and spring 2022 in a park and a forest habitat. D, darker cells indicate higher occurrence.

### Pathogens and behaviour

The composition of the community of pathogens in the rodent spleen were affected by behavioural types, but none of the behavioural variables did explain the richness of the pathogen community (Table 3). Richness of pathogens and ectoparasites combined was higher in *Apodemus* mice, compared to *Clethrionomys* voles, higher in males compared to females, and higher in the forest site compared to the park site (Table 3). Between forest rodent species, richness of the pathogens and ectoparasites combined was higher in *A. flavicollis* compared to *C. glareolus* (estimate = 0.41, *p* = 0.021), but not compared to *A. agrarius* (estimate = −0.10, *p* = 0.842). *C. glareolus* and *A. agrarius* did not differ (estimate = 0.32, *p* = 0.157) (Table A7).

**Table 3:** Pathogen richness with and without ectoparasites considered (A), pathogen community composition (B) in the spleen of forest rodents, with regard to rodent genus (*Apodemus* vs. *Clethrionomys*), sampling time (spring vs. autumn) and sampling site (forest vs. park site), sex (male vs. female) and animal behaviour, with pro. sections (proportion of floor sections covered in an open field test) coding for activity, and emergence from shelter in a dark-light test coding for boldness.

Data set, model	Factor						
	Genus	Season	Site	Sex	Pro. sections	Emergence	Explained Variance
A) Pathogen richness (Chi^2^)							
6 Pathogens	**4.06**	-	-	**3.93**	0.00	0.30	0.07
6 Pathogens + 2 ectoparasites	**7.58**	-	**5.95**	**4.44**	1.29	0.11	0.07
B) Pathogen community composition (R^2^)							
All species	**0.11**	**0.07**	0.00	0.02	**0.03**	0.01	0.24
*Apodemus* only	na	0.01	0.03	**0.10**	0.09	0.02	0.25
*C. glareolus* only	na	**0.20**	0.01	0.05	**0.07**	0.02	0.35

Models for pathogen richness were analysed via a GLM based on 93 individuals. Values represent likelihoods (Chi^2^ values). Effect sizes, errors, F values and p values can be found in supplemental table A2. Factors that did not significantly improve the model (AIC comparison < 2) were removed during model selection (marked with -), with the exception of activity and boldness measurements, our hypotheses, which were always retained in each model. Compositions of pathogen communities (B) were analysed via multivariate, permutational ANOVA based on 71 individuals, values refer to explained variance of the respective factor. Cells containing (na) indicate factors not relevant for the respective model. Bold letters indicate significant effect on the *p* < 0.05 level. For all models shown, the explained variances refer to the amount of variance explained by the entire model. For GLMs this corresponds to the R^2^ value of the respective model, while for the multivariate, permutational ANOVAs it refers to the sum of factor variances.

**Fig. 2:**
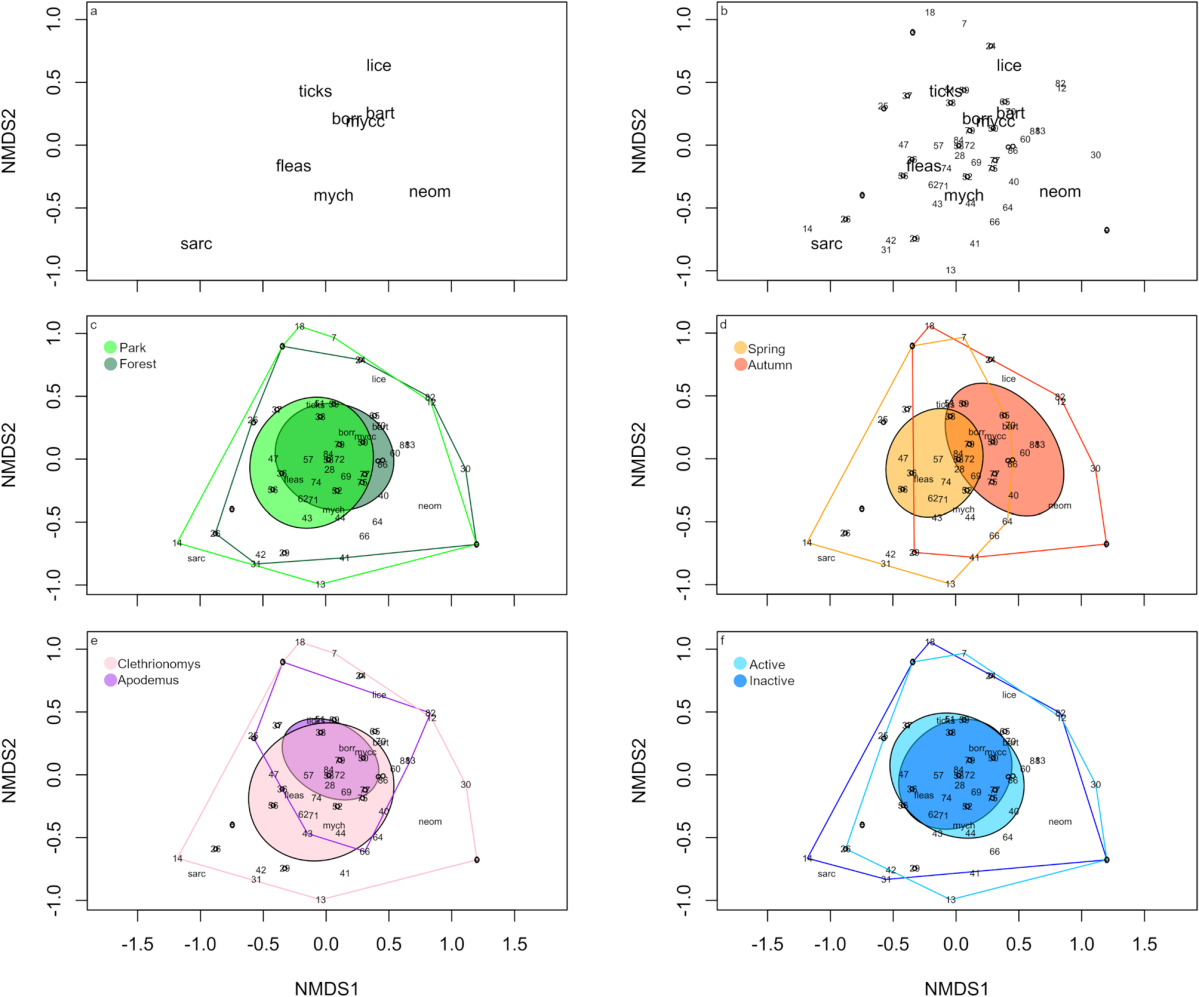
Ordination of combined pathogen and ectoparasite communities in 71 rodents from a forest and a park site (non-metric dimensional scaling plots (Stress = 0.054, clusters k = 4) (A) Pathogens found in the spleen (*Mycoplasma haemomuris (mych)*,* M. cocoides (mycc)*,* Bartonella (bart)*,* Candidatus Neoehrlichia (neom)*,* Sarcocystidae (sarc)*,* Borrelia (borr))* and ectoparasites found on the animal (fleas, lice and ticks). (B) Pathogen and Ectoparasite communities plotted together with rodent individuals (numbers), C-F) grouping of individuals by (C) sampling sites (D) sampling season, (E) host genus (Cle: *Clethrionomys*; Apo: *Apodemus*) and (F) by activity type.

The **composition** of the community of pathogens in the rodent spleen and ectoparasites (based on their occurrence data) differed among rodent genera (Permutational Multivariate Anova, F = 6.3, *p* = 0.001, Table 3B, Fig. 2E) explaining 11% of the variation, between the sampling times (F = 7.2, *p* = 0.002, 8% explained, Fig. 2D), and was related to the activity of the individual (F = 3.0, *p* = 0.046, explaining 3% of the variation). Within the pathogen community of each separate genus, activity explained 9% (*Apodemus*), or 7% (*Clethrionomys*), respectively (Fig. 2F, Table 3B). Boldness did not explain the pathogen community in either genus. In *Apodemus*, sex additionally explained 10% of the variance in the pathogen community composition. For *C. glareolus*, 20% of the variance in the pathogen community was explained by sampling season (F = 8.8, *p* = 0.001).

More active animals in both rodent genera were more likely to be infected with the most common pathogen, *Bartonella* sp. (33–65% of individuals per species infected, Table 2). *Sarcocystidae*, which occurred only in *C. glareolus*, were also positively related to activity, i.e., more active voles were also more likely to be infected (Table 4). Other pathogens were not affected by the activity of the individual (Table 4). The variable representing boldness did not have any effect on pathogen occurrence (Table 4).

**Table 4:** Overview of factors affecting single pathogen occurrence in the spleen of 93 forest rodents, with regard to rodent genus (*Apodemus* vs. *Clethrionomys*), sampling season (spring vs. autumn) and sampling site (site 1 (a forest) vs. site 2 (a park)), sex (female vs. male) and individual behavioural type (proportions of sections covered in an open field test is taken as a measure of activity, emergence from shelter in a dark light test was used as a measure of boldness).

Data set, model	Factor						
	Genus	Season	Site	Sex	Pro. sections	Emergence	Explained Variance
Single pathogen occurrence Ectoparasites							
Fleas	**3.60**	**13.16**	3.63	-	0.00	1.45	0.16
Ticks	**16.24**	2.20	**20.31**	3.00	0.63	**3.72**	0.29
Spleen bacteria							
Mych	3.34	-	-	**4.94**	0.00	0.39	0.08
Bart	2.83	**14.20**	-	**7.56**	**9.90**	0.01	0.23
Neom	-	-	**13.29**	-	1.28	0.13	0.15
Mycc (*Apodemus* only)	na	**5.97**	-	-	0.28	0.56	0.12
Borr (*Apodemus* only)	na	-	**6.26**	-	0.01	0.04	0.20
Sarc (*C. glareolus* only)	na	**8.15**	-	-	**3.40**	1.02	0.28

Occurrence of single pathogens was analysed with binomial, generalised linear models (GLM). Here we provide likelihoods (Chi^2^ values) for a comparative overview (bold effects indicated *p* < 0.05). Effect sizes, errors, F values and p values can be found in supplemental table A3. Factors that did not significantly improve the model (AIC comparison < 2) were removed (marked with -), with the exception of activity and boldness measurements, our hypotheses, which were retained in each model. Cells containing (na) indicate factors not relevant for the respective models. For all models shown, the explained variances refer to the amount of variance explained by the entire model (R^2^ value). Pathogens occurring predominantly within one host genus (i.e. <10% of individuals of the other genus were infected) were analysed within the respective taxon (*Mycoplasma coccoides* (Mycc) and *Borrelia* (Borr) only in mice), *Sarcocystidae* (Sarc) only in voles).

 The probability for a rodent to be infested by ectoparasites was influenced by individual behaviour, and differed among species and genera (Table 4 and A6, A7). More *A. agrarius* individuals had fleas compared to *C. glareolus* (effect estimate = 2.19, *p* = 0.019) and tended to have also more fleas compared to *A. flavicollis* (estimate = 2.04, *p* = 0.058) individuals. *C. glareolus* and *A. flavicollis* did not differ regarding their flea occurrence (estimate = 0.15, *p* = 0.971) but regarding their tick occurrence, with more *A. flavicollis* having ticks compared to *C. glareolus* (estimate = 3.75, *p* = 0.003; Table A7). Tick occurrence did not differ between the two *Apodemus* species (estimate = −2.15, *p* = 0.172) but tended to differ between *A.agrarius* and *C. glareolus* (estimate = 1.60, *p* = 0.091; Table A7). For all rodents species, tick occurrence was positively correlated to emergence, i.e., the longer it took until the animals’ head appeared in the opening (the longer = the shyer), the more likely it was that ticks were found (Table 4).

Occurrence of many ectoparasites and pathogens (i.e., fleas and ticks, *Candidatus Neoehrlichia mikurensis* and ***Borrelia sp.***) was higher on the forest site, compared to the park (Table 4, Table A6). Differences between males and females were observed in the occurrence of *Mycoplasma haemomuris*, and *Bartonella* sp., with males having higher occurrences of the pathogens than females in both cases (Table 4). *Sarcocystidae* and *Borrelia* were rare in *Apodemus* (2–12%, depending on species). In *C. glareolus*, *Sarcocystidae* was found only during spring (10 cases; Estimate = 2.62, *p* = 0.015), and in *Apodemus*, *Borrelia* were only found at the forest site (5 cases, Table 2; Estimate = 2.24, *p* = 0.046; Table A3).

## Discussion

Understanding the impact of host behaviour on infection patterns in wild reservoirs hosts is an important step towards understanding disease dynamics, which can have direct benefits for public health by improving epidemiological surveillance and disease management strategies. Thereby it is important to consider that individual behaviours of hosts may interact to influence the infection risk for multiple pathogens simultaneously, rather than affecting each pathogen individually. Here we applied this integrative framework by quantifying two different individual behavioural traits, and investigating how these affect pathogens found in the spleen, as well as ectoparasites of two rodent genera. We found that boldness only influenced the probability of individuals being infested by ticks, while individual activity affected community composition of pathogens as well as the probability of individuals being infected with *Sarcocystidae* and *Bartonella*. Although most variation in infection patterns was explained by host genus, season or sampling site we could still show that animal personality contributes to the distribution and prevalence of pathogens in wild rodents.

Pathogens found in this study are typical for rodents inhabiting forests in Europe: *Bartonella* can be pathogenic on both rodents and humans^34,35^–^36^, *Mycoplasma* is a common parasite of mammals^37^ including *Apodemus* mice and *C.glarolus*^38^^39^. *M. haemomuris* can cause infectious anaemia in rodents^40^. We did not find support for associations of Mycoplasma to behavioural traits. *Mycoplasmae* are among the smallest self-replicating prokaryotes^37^ and can spread airborne for many kilometres^41^.*Candidatus Neoehrlichia* sp., was present in ~ 25% of all individuals in our study, matching earlier findings^42,43^–^44^. Although related *Neoehrlichia mikurensis* causes inflammatory disease in humans, infected rodents seem asymptomatic^44^. *Neoehrlichia sp.* can be transmitted by ticks^45^, however the probability to have ectoparasites was not related to the infection in our data. Further, *Borrelia* was not related to behaviour, but we have to be careful with this genus since the spleen is not the target organ to detect *Borrelia* infections.

We found that *Bartonella* had higher levels of prevalence in more active individuals, and in males. Bartonella seems to be abundant in rodents and their fleas^46^. High activity levels may increase encounters with conspecifics and their fleas, and thus put individuals at a higher risk of contracting *Bartonella*.

This finding is in accordance with sex differences we detected in both *Bartonella* and *Mycoplasma*, with a higher occurrence in males than in females. Males in most mammals have larger home ranges and more interactions with conspecifics than females, and often also carry higher flea loads (e.g^47^), which increases the risk of *Bartonella* contraction. Meanwhile, in a study on Microtus voles, *Bartonella* infections were not linked to direct contact rates^20^, indicating that individual activity may translate to other behaviours, e.g. space use or the use of joint nests or points of interest, where fleas can be contracted. However, while the probability to be inflected with Bartonella was dependent on individual activity, the probability to be infested with fleas was not influenced by activity in our study. This suggests that there is no influence of animal personality on the likelihood of encountering fleas and that the latter is also independent of exposure to *Bartonella*. Thus, the positive association between *Bartonella* and individual activity might not reflect differences in encounter rates with fleas, but rather individual variation in host susceptibility. This would align with the findings of Koprivnikar et al.^48^ who could show an association between a behavioural syndrome, formed by activity, boldness and exploration, and the susceptibility to parasite infection in tadpoles.

Ectoparasite infestation can positively correlate with personality traits, for example with boldness in ground squirrels^4^^5^, and exploration in great tits^49^. However, encounter probability with ticks and other ectoparasites might not always be directly linked to personality traits but could be indirectly resulting from personality-dependent social interactions, or space use. For example, bolder *C. glareolus* and *A. agrarius* had larger home ranges with higher levels of ground cover (i.e., vegetation cover in 10 cm from the ground) compared to shyer conspecifics^18^^19^. These personality-dependent microhabitat preferences could in turn affect encounter rates with ticks, as the tick larva, which preferably infest rodents, occur primarily in lower vegetation layers (0–9 cm^50^). In great tits (*Parus major*) more explorative individuals had more social associations compared to their less explorative counterparts^51^ which could influence parasite encounter rates. Higher numbers of contacts to conspecifics might translate to higher chances of encountering with parasites infested individuals or areas where parasites occur, as it was shown for sleepy lizards (*Tiliqua rugosa*) where bolder individuals had more social interactions and a higher probability to be infested with ticks^52^.

In our study shyer rodent individuals surprisingly had a higher probability to be infested by ticks compared to bolder individuals, which was also found in an earlier study on *C. glareolus* in Sweden (Erixon et al. submitted). This might be correlated to the finding that shyer rodents use areas of higher maximum vegetation height, i.e., areas characterized by bushes and trees^18^. Such areas might be more attractive habitats for some local ticks, such as *Ixodes Ricinus*, and thus, might represent areas with higher tick abundances^53^. The overlap of areas preferred by shy individuals with areas of higher tick abundances might result in higher chances of encounters between hosts and parasite and might explain the observed pattern of probability of tick infestation between bold and shy rodents. Rather than just vegetation density, vegetation types might be also relevant for the encounter probability between ticks and hosts species in a certain area, as well as species-specific habitat preferences of the parasites. Since ticks were not determined to species in our study, we cannot say anything regarding species specific effects, but it seems likely that different tick species might occur in some habitat areas in higher numbers compared to other areas. The specific relationship between boldness and tick infestation probability might thus be a result of species-specific non-random distribution patterns of ticks paired with non-random, personality driven distribution patterns of the host species. Depending on the species observed the overlap of these patterns might change explaining why contradictory findings for the effect of boldness on tick infestation probability exist.

Animal personality traits have been shown to correlate with individual space use^54^, spatial overlap with conspecifics and heterospecifics (e.g^18,19^), and fitness proxies (e.g^13^) and can significantly affect the likelihood of an individual contracting infections. It is known that a small proportion of individuals contribute disproportionately to the transmission of a pathogen^15^ nowadays called “superspreader”. Both sociability and boldness have been associated to this asymmetry of transmission, as well as activity and exploration. Infection patterns have often been reported to be biased towards males^55^ but when considering personality and sex together patterns are less clear. For little brown bats it was for example shown that the bias in parasite infection towards one sex changed depending on the parasite species. The positive effect of exploration on the infection probability on the other hand was the same in both sexes^56^. Thus, the asymmetry of infection related to the interaction of sex and personality traits might be highly pathogen/parasite specific. Parasitized individuals may differ in their approach to predators (e.g^57–59^.) or the general level of activity^60,61^–^62^ but it is often unclear whether the differences are due to behavioural changes induced by the pathogen, or were the pre-condition to contract pathogens. The rodents in our study were more likely to carry *Bartonella spp* if they were more active. Effects of activity were picked up for pathogen composition (Table 3A), however the community effect may have been largely driven by the occurrence of *Bartonella* in both genera, and the occurrence of Sactocystes in voles.

## Conclusion

Here, we demonstrate that animal personality traits can contribute to the distribution, prevalence, and co-occurrence of pathogens in wild rodents. Active individuals carried more pathogen species and a different pathogen community then less active individuals. Hosting multiple pathogens simultaneously may impose increased immune demands, potentially resulting in trade-offs or immune suppression that further influence susceptibility to additional infections. These findings highlight the importance of incorporating individual behavioural variation into epidemiological frameworks and disease management strategies.

## Methods

### Rodents, sites and capture protocol

Rodents were sampled in October 2021 and May 2022. We collected both behavioural and pathogen data from 99 individuals, belonging to the species bank vole *Clethrionomys glareolus* (46 individuals), yellow necked mouse *Apodemus flavicollis* (26), striped field mouse *Apodemus agrarius* (21), and common vole *Microtus arvalis* (6). The latter was excluded from the analyses presented here (*n* = 93, two genera) due to small sample size. Sampling was replicated at two sites, a botanical garden in a semi-forested, urban park (290 ha) in Potsdam, Brandenburg, northeast Germany, surrounded by urban settlements with a mixture of sealed and wooded areas and a constant human presence (park site); and a forested area at the edge of a large forest (875 ha), 3 km distant from the first site and composed of mixed coniferous forests, meadows, train tracks and a main road, but no walking paths (forest site). Details of the area and rodent capture methods are described in Firozpoor et al.^63^. Trapping was conducted on both sites in each sampling time. In short, we captured, marked and released rodents for a week conducting behavioural tests, and the next week we collected and sacrificed these individuals for the pathogen sampling. In total we conducted 164 animal personality tests (206 in total, of which 19% were second and 4% third tests). Some individuals were tested but not re-captured when collecting the individuals for pathogen sampling, but we included their behavioural data to describe the variability and repeatability of the behavioural data collection.

Rodents were captured using a combination of live traps (*Longworth and Ugglan* traps) with a 1 cm hole to allow shrews to escape^64^ since these were not target species. At each site a total of 100 inactive traps were placed in 4 transects with 25 m spacing among transects and 10 m between traps and, pre-baited for 2–3 days. After pre-baiting, traps were equipped with fresh bait and nesting material and activated in the evening. The following morning empty traps were deactivated. Captured animals were kept in their traps and provided with fresh food and nesting materials. Lactating and pregnant females were not included in the testing and were directly released on the capture location. The other captured animals were transported in their trap up to 100 m from their point of capture to the behavioural testing arena at a shady location on the site and tested during the morning. After the test, animals were weighed and individually fur-marked using a small scissor, and subsequently released at the location of capture during the first week, or euthanised during the second week. The proportion of animals tested repeatedly varied among the species (25% of *C. glareolus*, 39% of *A.agrarius*, and 29% of *A. flavicollis* had repeated tests).

### Behavioural testing

We combined two standard tests, the dark-light and the open-field test^18^. Both tests were video recorded from above using Cam Park Action Cameras X15 and scored afterwards. The dark-light test measures the willingness of an individual to leave a dark protected shelter to enter an open, well-lit and potentially dangerous new space^65^. The setup consisted of an opaque PVC tube (length 32 cm, diameter 10.5 cm, closed by a slightly smaller cylinder) connected to a plastic arena that emulated an exposed area (Figure A1 A and B). The entrance leading to the arena had an outer opaque door and an inner, transparent door. The latter was designed as a one-way, self-closing door only opening towards the arena and closing once the individual passed it, thereby preventing re-entering of the tube (Figure A1 C). Rodents were first brought inside the PVC tube and left to acclimatize in the dark for a minute. Afterwards the outer door leading to the arena was opened, letting light in, and a 300 s (s) timer started. The animal now could either stay in the tube or push the inner door and enter the arena. Two latencies were recorded during this test: *latency head* describes the elapsed time until the animal first peaked its head until the neck into the arena. The *Latency body* describes the time until the animal enters the arena with its full body excluding the tail and concludes the dark-light test. If the rodent did not leave the tube, the test concluded at the 300 s mark, and the rodent was assigned a latency of 300 s for both measurements. In this case, the rodent was carefully displaced by the operator into the arena, either by slowly rotating the entrance tube (first step), or by gradually reducing the space in the tube by slowly pushing the smaller cylinder into the tube (second step).

As soon as the animal had entered the arena with its full body, the open-field test started. This test measures the exploratory behaviour and the general activity of an animal within a delimited open space^66^^67^. We used a circular arena with a diameter of 120 cm surrounded by 60 cm high walls and a net on top. Drawings on the arena bottom divided the arena into an inner (45 cm wide) and outer (15 cm wide) section, and 8 sectors, meeting running through the center, further divided the arena into 16 sections. The test ran for 300 s. During this time, five measurements were recorded: (1) *Latency center*, which describes the time in seconds until the animal first steps into the inner circle on its own initiative, with its full body excluding the tail, and it defaults to 300 s if this condition is not met (cases where the animals fled the dark-light entrance tube when we tried to displace it carefully, and thereby crossed the line, were not counted). (2) The proportion of visited *sections* with the full body excluding the tail. (3) The frequency of *jumps* (total number of jumps/5 min) (4) The *crossing* frequency (total number of crossings into the inner ring/5 minutes). (5) Every ten seconds *activity* was recorded as a binary value, wherein the animal was currently active or inactive. A rodent was defined as active if any type of locomotion was displayed.

### Pathogen and ectoparasite detection

 The week following personality tests, traps were activated at the same locations every evening, and checked daily for a week. Captured rodents were transported inside of their traps to the Animal Ecology Institute of the University of Potsdam. Rodents were euthanized through cervical dislocation without sedation, which is standard for small birds and rodents < 150 g body mass, in accordance with German Regulations on the protection of animals used for scientific purposes (TierSchVersV, Appendix 2). Before dissection, weights (Table A9) and morphological features of the dead rodent were recorded and a thorough collection of ectoparasites was performed for a total of five minutes using a lice comb and tweezers, conserved in 0.5 ml Eppendorf tubes filled with Ethanol 70%. Ectoparasites were assigned to three functional (and taxonomic) groups: ticks, lice, and fleas, using a stereo microscope. Rodent dissections were performed according to the protocol described in Herbreteau et al.^68^, for field and laboratory rodent studies. Several organs were collected and stored for PanEuropean collaborative studies on SARS-CoV-2 distribution in rodents^69^ and other rodent-borne diseases^70^.

The spleen is a phagocytic filter that removes bacteria from the bloodstream and it is an antibody-producing organ^71^ so that recent bacterial infections can be detected. Spleen samples were kept in Ethanol 96% at 4 °C until analysis. To identify current or very recent bacterial infections, a 16 S rRNA gene amplicon sequencing was employed on rodent spleens. DNA extraction, PCR amplification, and other necessary steps were followed as detailed in Galan et al.^72^. In brief, each DNA extraction was analysed in two independent replicates. Three MiSeq sequencing runs were performed and the raw data are publicly available. Bacterial taxa (or parasitic taxa including Sarcocystidae) are reported as clusters, or operational taxonomic units (OTUs), are sequences that share enough similarity in the molecular level to be sorted together. OTUs were taxonomically classified using Basic Local Alignment Search Tool (BLAST) and the Silva database v138.1 to infer species or genus identity where possible.

### Data and statistics

 To investigate effects of behaviour on pathogen occurrence, we first had to identify behavioural variables that quantified consistent, inter-individual differences in behaviour^73^^74^. For this we calculated a repeatability score *R* for each separate variable obtained in the behavioural tests in a mixed-effect models framework^75^, where a value *R* is obtained as the proportion of the total variance accounted for by differences among individuals and the total variation, with the package *rptR*^75^. Repeatability analyses were based on 186 individuals tested in the behavioural tests, with 57 of those individuals being tested repeatedly (20 *C. glareolus*, 22 *A.agrarius*, 15 *A. flavicollis*). Models calculating repeatabilities always had the same structure, with the respective behavioural variable measured in the behavioural test as a response variable and only including random intercepts (individual ID) but no additional fixed factors. The appropriated error structures were calculated by considering the respective underlying distribution of each behavioural variable (Table A1). The higher the *R* and the more skewed away from 0 the confidence interval was, the more repeatable the particular behaviour.

*C. glareolus* showed the highest amount of repeatable behaviour, while in both *Apodemus* species most behavioural variables were not repeatable (Table A1). Only the “proportion of sections” covered in the open field test was repeatable for all three species. Thus, we refrained from using combined scores as a quantitative measure of personality traits, instead we used the “proportion of sections covered” directly as an indicator of individual activity levels. Further, since the number of individuals with repeated tests was rather small, we did not calculate a personality score per animal that would account for learning or habituation in repeated testing, but we used the values obtained in the first testing round of each animal. The latency to stick the head out of the tube, “latency head” was used as a quantitative measure for boldness, even though it was repeatable only for *C. glareolus* in this study (Table A1), but in many earlier studies with larger sample sizes, this variable has been shown to be repeatable also for different *Apodemus* species^19^^76^^77^. Due to its bimodal distribution (not sticking the head out at all, versus sticking the head out during the test) it was converted into a binary variable and called “emergence”. Binary variables showed the same repeatability patterns as the original variables (Table A1). The proportion of sections covered and emergence negatively correlated (Spearman correlation; S = 3285817, *p* = 0.001, Rho = −0.20; Fig. 1), but weakly enough^78^^79^, to use both variables as covariates in subsequent statistical models.

 Preliminary analysis (supplement tables A4 – A8) showed no difference between the two *Apodemus* species, neither for behavioural variables nor for the effect of behavioural variables on pathogen and ectoparasite richness, community composition and occurrence, but both mice species differed from *C. glareolus*. Repeatabilities of behavioural variables differed between the single *Apodemus* species compared to the data set of both *Apodemus* species combined (Table A1). These differences are most likely due to the differences in sample size and number of repeatedly tested individuals, as both crucially influence the repeatability estimations^80^. Based on these findings, and considering that pooling the data on mice would allow to even out the sample size differences between species, data from the two *Apodemus* species was combined for all subsequent analyses resulting in the presented comparisons referring to the genus, rather than the species level.

To understand how sampling season, site, genus, sex and test repeat contributed to the variance observed in the chosen **behavioural variables**, we used them as fixed factors in generalised linear mixed models (GLMM) with either emergence or proportion of sections, as a response variable. Individual ID was always incorporated as a random factor. For both variables mixed models including all individuals tested (*n* = 186) were calculated considering a binomial distribution with a logit link function to model the appropriate error structure of the data. GLMMs were calculated with the package *lme4*^81^.

Alpha **diversity** of pathogens and ectoparasites within animals was quantified using *richness*. We calculated two different models, one only including data from pathogens found in the spleen and one considering data on pathogens and ectoparasites together. Both models were based on 93 individuals that had data for both, the behavioural test and the pathogens and ectoparasites. We chose to calculate these two separate models because the level of accuracy and repeatability of pathogen prevalence in the spleen is probably higher than of the ectoparasite counts that are extremely variable in time. We refrained from doing a separate analysis on just the ectoparasites alone, since only two taxa with sufficient data were obtained. Further, since some of the ectoparasites are transmitting the pathogens we investigated, analysing them together seemed reasonable.

To analyse if **richness** was affected by sampling time, sampling site, genus, sex or the behavioural measures, we incorporated them as fixed factors into a GLM, with either pathogen richness, or combined pathogen and ectoparasite richness as the response variables (Table 3). Since richness is a count variable, we fitted statistical models with poisson distributions.

**Community** **composition** analyses (Permutations and ordinations) required the removal of data from samples without any pathogen detection, and the removal of pathogens with less than 10% of cases infected. Thus, 71 individual samples were included to the analyses on pathogen communities (35 *C. glareolus*, 14 *A. agrarius* and 22 *A. flavicollis)*.

To quantify the variance explained by behaviour on the community compositions of pathogens, we used permutational multivariate ANOVA (adonis2 command) in the *vegan* package^82^ adding the two behavioural measures, sampling time, site, genus and sex as explanatory fixed factors. Three different permutational multivariate ANOVAs were conducted. The first one was run with data on pathogens and ectoparasites found in mice and voles, while the other two analyses were based on either data for just voles or just mice respectively. This subdivision of the data was done because mice and voles differed in the occurrence of pathogens, with some pathogens being sufficiently present (> 10% of animals infected) in only one of the genera but not the other (Tables 2 and 3). Thereby subsetting the data allowed to look at genera specific patterns of pathogen communities and how they are influenced by behavioural measures, sampling time, site and sex.

To test the effects of sampling time, sampling site, genus, sex or the behavioural measures on **single pathogen and ectoparasite occurrences**, we used them as fixed factors in separate GLMs for each pathogen/ectoparasite type (Table 4). Occurrence (yes/no) of the respective pathogen and ectoparasite was used as binary response variable and a binomial distribution was assumed for all occurrence GLMs. 

In each analysis we always included the two behavioural variables as fixed factors, to test our behaviour related hypotheses. We challenged non-target covariates, i. e. genus, site, sex and season. If a covariate was improving the model fit (AIC comparison, delta AIC > 2), or if its effect on the respective response was significant, we kept it in the model, otherwise we removed it.

### Ethics declarations

Collection of animals was permitted by the Landesamt für Umwelt (LFU-N4-4730/11 + 10#120786/2021), capture and testing methods were permitted by the Landesamt für Arbeitsschutz, Verbraucherschutz und Gesundheit (2347-A-16-1-2020). The study complies with the applicable international, national, and/or institutional guidelines for the use of animals and with the ASAB/ABS Guidelines for the Use of Animals in Research and authors complied with the ARRIVE guidelines.

## Supplementary Information

Below is the link to the electronic supplementary material.

Supplementary Material 1

## Data Availability

Data from the thee MiSeq sequencing runs is available on Zenodo ([https://doi.org/10.5281/zenodo.12518285]: Run14, Run186 and Run2018).The datasets for the behavioural analyses are available from the corresponding author on reasonable request.
