# Supplementary material for: Individual activity of forest rodents correlates to pathogen communities
Source: Sci Rep. 2026 May 9;16:14684. doi: 10.1038/s41598-026-51276-6 (PMC13157493; doi:10.1038/s41598-026-51276-6)
Supplement: Supplementary file 1 — Supplementary Material 1 [file 41598_2026_51276_MOESM1_ESM.pdf]

Individual activity of forest rodents correlates to pathogen communities

Jana Eccard, Jasmin Firozpoor, Mario Escobar, Maxime Galan, Nathalie Charbonnel

Table A1: Repeatability of original behavioural variables from an emergence test (dark-light) and an open field test for three species of forest rodents (*R*: repeatability, SE: Standard Error of Repeatability, CI: Confidence interval and *p*: significance level) and the genus *Apodemus* (both species combined). Emergence is a binary representation of latency head, since for many animals the head did not emerge within 300 seconds (Figure 1). Repeatability analyses were based on 186 individuals tested in the behavioural tests (79 *C. glareolus*, 56 *A.agrarius*, 51 *A. flavicollis*).

Variable	Family	R	SE	CI low	CI high	p	
latency head	gaussian	**0.40**	**0.16**	**0.01**	**0.67**	**0.013**	*Clethrionomys glareolus*
emergence	binomial	0.24	0.22	0.00	0.99	0.062	
latency body	gaussian	0.17	0.18	0.00	0.98	0.120	
latency center	gaussian	0.11	0.08	0.00	0.27	0.195	
jumps	poisson	0.00	0.12	0.00	0.40	0.500	
crossings	poisson	**0.57**	**0.14**	**0.23**	**0.79**	**0.003**	
no. sections	poisson	**0.36**	**0.22**	**0.00**	**0.75**	**0.020**	
activity	gaussian	**0.70**	**0.09**	**0.50**	**0.84**	**<0.001**	
latency head	gaussian	0.00	0.12	0.00	0.38	0.500	*Apodemus agrarius*
emergence	binomial	0.01	0.10	0.00	0.25	0.471	
latency body	gaussian	0.01	0.11	0.00	0.28	0.471	
latency center	gaussian	0.04	0.15	0.00	0.42	0.384	
jumps	poisson	0.00	0.13	0.00	0.46	1.000	
crossings	poisson	0.00	0.12	0.00	0.38	1.000	
no. sections	poisson	**0.36**	**0.22**	**0.00**	**0.73**	**0.030**	
activity	gaussian	0.12	0.15	0.00	0.51	0.257	
latency head	gaussian	0.18	0.19	0.00	0.62	0.216	*Apodemus flavicollis*
emergence	binomial	0.19	0.42	0.00	0.99	0.192	
latency body	gaussian	**0.98**	**0.07**	**0.98**	**1.00**	**<0.001**	
latency center	gaussian	0.00	0.37	0.00	0.99	0.500	
jumps	poisson	0.19	0.20	0.00	0.65	0.262	
crossings	poisson	0.00	0.15	0.00	0.56	0.500	
no. sections	poisson	**0.36**	**0.27**	**0.00**	**0.84**	**0.020**	
activity	gaussian	0.00	0.15	0.00	0.48	1.000	
latency head	gaussian	0.08	0.12	0.00	0.38	0.323	*Apodemus*
emergence	binomial	0.05	0.14	0.00	0.29	0.314	
latency body	gaussian	0.09	0.10	0.00	0.27	0.215	
latency center	gaussian	0.00	0.14	0.00	0.25	0.492	
jumps	poisson	**0.32**	**0.16**	**0.00**	**0.60**	**0.036**	
crossings	poisson	0.01	0.10	0.00	0.33	0.480	
no. sections	poisson	**0.36**	**0.18**	**0.00**	**0.70**	**0.010**	
activity	gaussian	0.03	0.10	0.00	0.33	0.420	

Table A2: Estimated pathogen richness in spleens of 96 rodents, using A) 6 pathogens or B) 6 pathogens and 2 ectoparasites (compare to manuscript table 3). Factors include rodent genus (Apodemus vs Clethrionomys), sampling season (autumn vs spring) and sampling site (park vs forest), sex (female vs male) and animal behaviour. Pathogen richness was analysed with generalised linear model using a poisson distribution. Values represent coefficient-level tests for factor estimates (Wald tests), standard errors for the estimates, z- and p values, as well as the explained variance, specified error distribution and AIC value of the calculated models. For categorical factor estimates refer to the factor level indicated in brackets compared to the respective reference level. Factors that did not significantly improve the model (AIC comparison < 2) were removed during model selection (marked with -), with the exception of activity and boldness measurements, our hypotheses, which were always retained in each model. Bold letters indicate significant effect on the p<0.05 level.

Factors (Factor level)	Estimate	SE	Z value	P value	Explained variance	Error distribution	AIC
**A) Spleen pathogen richness**							
Intercept	0.72	0.47	1.53	0.125	0.07	poisson	212.24
Genus (Cle)	**-0.40**	**0.20**	**-1.99**	**0.047**			
Season (spring)	-	-	-	-			
Site (forest)	-	-	-	-			
Sex (male)	0.39	0.20	1.94	0.053			
Pro. sections	-0.01	0.46	-0.01	0.990			
Emergence	-0.10	0.19	-0.54	0.588			
**B) Pathogen + Ectoparasite richness**							
Intercept	0.56	0.37	1.51	0.131	0.07	poisson	310.76
Genus (Cle)	**-0.37**	**0.14**	**-2.73**	**0.006**			
Season (spring)	-	-	-	-			
Site (forest)	**0.31**	**0.13**	**2.41**	**0.016**			
Sex (male)	**0.30**	**0.15**	**2.07**	**0.039**			
Pro. sections	0.39	0.35	1.10	0.270			
Emergence	0.05	0.14	0.33	0.742			

Table A3: Single pathogen and ectoparasite occurrence in 93 forest rodents with regard to rodent genus (Apodemus vs Clethrionomys), sampling time (autumn vs spring) and sampling site (park vs. forest), sex (female vs male) and individual behavioural type (proportions of sections covered in an open field test is taken as a measure of activity, latency until sticking out the head in a dark light test was used as a measure of boldness). Occurrence of single pathogens and ectoparasites was analysed with binomial, generalised linear models (GLM). Values represent coefficient-level tests for factor estimates (Wald tests), standard errors for the estimates, z- and p values, as well as the explained variance, specified error distribution and AIC value of the calculated models. For categorical factor estimates refer to the factor level indicated in brackets compared to the respective reference level. Factors that did not significantly improve the model (AIC comparison < 2) were removed (marked with -), with the exception of activity and boldness measurements, our hypotheses, which were retained in each model. Empty cells indicate factors not being included in initial models. Pathogens occurring predominantly within one host genus (i.e. <10% of individuals of the other genus were infected) were analysed within the respective taxon (*Mycoplasma coccoides* (Mycc) and *Borrelia* (Borr) only in mice), *Sarcocystidae* (Sarc) only in voles). Models on *Borrelia* and *Sarcocystidae* occurrences were calculated as Firth's logistic regressions, adding a penalty term to the models' likelihood functions to account for total separation of outcomes of the binary response variables between season or study sites.

	Factors (Reference level)	Estimate	SE	z value	p value	Explained variance	Error distribution	AIC
Ectoparasites	**Fleas**							
	Intercept	-1.03	1.36	-0.76	0.447	0.16	binomial	111.98
	Genus (Cle)	-1.00	0.54	-1.85	0.064			
	Season (spring)	**1.98**	**0.58**	**3.40**	**0.001**			
	Site (forest)	1.04	0.57	1.84	0.066			
	Sex (male)	-	-	-	-			
	Pro. sections	0.06	1.26	0.05	0.960			
	Emergence	0.65	0.55	1.19	0.235			
	**Ticks**							
	Intercept	-2.45	1.52	-1.61	0.107	0.29	binomial	97.61
	Genus (Cle)	**-2.45**	**0.69**	**-3.56**	**<0.001**			
	Season (spring)	1.01	0.69	1.47	0.142			
	Site (forest)	**2.72**	**0.70**	**3.88**	**<0.001**			
	Sex (male)	1.08	0.64	1.71	0.088			
	Pro. sections	1.03	1.29	0.80	0.426			
	Emergence	1.16	0.63	1.85	0.065			
Spleen bacteria	**Mych**							
	Intercept	-0.47	0.18	-0.40	0.691	0.08	binomial	128.32
	Genus (Cle)	-0.87	0.48	-1.80	0.071			
	Season (spring)	-	-	-	-			
	Site (forest)	-	-	-	-			
	Sex (male)	**1.06**	**0.49**	**2.18**	**0.030**			
	Pro. sections	0.30	0.49	0.62	0.533			
	Emergence	-0.02	1.13	-0.02	0.983			

**Bart**							
Intercept	**-4.73**	**2.32**	**-2.04**	**0.042**	0.22	binomial	111.00
Genus (Cle)	-0.89	0.54	-1.66	0.098			
Season (spring)	**-2.26**	**0.68**	**-3.32**	**0.001**			
Site (forest)	-	-	-	-			
Sex (male)	**1.56**	**0.61**	**2.55**	**0.011**			
Pro. sections	**5.97**	**2.50**	**2.39**	**0.017**			
Emergence	-0.05	0.53	-0.09	0.926			
**Neom**							
Intercept	-1.34	1.30	-1.04	0.301	0.15	binomial	102.07
Genus (Cle)	-	-	-	-			
Season (spring)	-	-	-	-			
Site (forest)	**2.07**	**0.66**	**3.12**	**0.002**			
Sex (male)	-	-	-	-			
Pro. sections	-1.31	1.18	-1.11	0.269			
Emergence	0.18	0.52	0.36	0.723			
**Mycc (Apodemus only)**							
Intercept	0.37	2.73	0.14	0.892	0.12	binomial	65.42
Genus (Cle)							
Season (spring)	**-1.93**	**0.89**	**-2.17**	**0.030**			
Site (forest)	-	-	-	-			
Sex (male)	-	-	-	-			
Pro. sections	1.52	2.92	0.52	0.603			
Emergence	-0.55	0.74	-0.74	0.459			
**Borr (Apodemus only)**							
Intercept	-2.54	3.58	na	0.498	0.20	binomial	10.02
Genus (Cle)							
Season (spring)	-	-	-	-			
Site (forest)	**2.24**	**1.33**	**na**	**0.046**			
Sex (male)	-	-	-	-			
Pro. sections	-1.03	3.56	na	0.794			
Emergence	-0.03	1.04	na	0.981			
**Sarc (C.glareolus only)**							
Intercept	-5.34	2.65	na	0.008	0.28	binomial	14.54
Genus (Cle)							
Season (spring)	**2.62**	**1.41**	**na**	**0.015**			
Site (forest)	-	-	-	-			
Sex (male)	-	-	-	-			
Pro. sections	2.64	2.46	na	0.226			
Emergence	-0.60	0.73	na	0.431			

Table A4: Results of generalized linear mixed models of the two behavioural variables, considering species differences instead of differences between genera. Shown are factor estimates of the fixed effects, the standard error (SE) of the fixed effects as well as the t-value. Additionally, the amount of variance explained by the fixed effects alone (marginal R squared) and the fixed and random effects together (conditional R squared) and the AIC value are shown. Significant effects are highlighted in bold.

	**Emergence**				**Proportions sections**			
	**Estimate**	**SE**	**z value**	**Pr(>|z|)**	**Estimate**	**SE**	**z value**	**Pr(>|z|)**
(Intercept)	**11.41**	**2.88**	**3.96**	**< 0.001**	1.55	0.80	1.94	0.052
Season (spring)	0.12	1.89	0.07	0.948	-0.45	0.57	-0.79	0.432
Site (forest)	-0.87	2.06	-0.42	0.673	0.73	0.65	1.13	0.259
Species (Aagr)	**-22.50**	**2.91**	**-7.74**	**< 0.001**	0.81	0.56	1.45	0.147
Species (Afla)	**-22.76**	**3.09**	**-7.36**	**< 0.001**	1.48	0.85	1.74	0.082
Repeat	0.17	1.29	0.14	0.893	0.23	0.50	0.47	0.641
Marginal R^2	0.05				0.16			
Conditional R^2	1.00				0.16			
AIC	155.04				128.64			

Table A5: Post hoc comparison for differences between the species for emergence. Significant effects are highlighted in bold.

	**Value**	**SE**	**Df**	**Chisq**	**Pr(>Chisq)**
Cgla-Aagr	1	2.91	1	59.82	**<0.001**
Cgla-Afla	1	3.10	1	54.09	**<0.001**
Aagr-Afla	0.56	2.52	1	0.01	0.918

Table A6: Statistical models for single pathogens (A) and pathogen communities (B,C) with regard to rodent species (*Apodemus agrarius, A. flavicollis and Clethrionomys glareolus*), season (Spring vs Autumn) and sampling site (Forest site vs Park site), sex (Male vs Female) and animal personality. We used single variables measured in the open field tests directly after capture (see text). Occurrence of single pathogens (A) was analysed with binomial, generalised linear models (GLM) and likelihoods (Chi2) are given. Communities were analysed for richness (B) with glm and poisson family. Factors that did not significantly improve the models in A and B (AIC comparison) were removed (marked with -), with the exception of activity and boldness measurements, our hypotheses, retained in each model. Composition of the pathogen community (C) was analysed with multivariate, permutational Anova, here we present the explained variance per factor tested with Chi^2. Pathogens with less than 10% of animal infected (compare Table 2) were removed from the respective analyses, and some analyses and communities had to be restricted to one taxon (compare Table 2). Significant effects significance levels (<0.05). are highlighted in bold.

**Data set, model**	**Factor**						
	**Species**	**Season**	**Site**	**Sex**	**Pro. sections**	**Emergence**	**Explained Variance**
**A) Single pathogen occurrence Ectoparasites** (Chi2, bold if significant)							
Fleas	**9.51**	**14.77**	**7.22**	-	0.38	1.35	0.21
Ticks	**20.49**	1.61	**12.59**	3.51	0.22	3.46	0.32
**Spleen bacteria**							
Mych	-	-	-	**7.12**	0.02	0.00	0.10
Bart	4.25	**14.22**	-	**8.39**	**7.36**	0.04	0.24
Neom	5.55	3.05	**6.34**	2.27	2.37	0.76	0.28
Mycc (Apodemus only)	-	**5.97**	-	-	0.28	0.56	0.12
Borr (Apodemus only)	-		**6.26**	-	0.01	0.04	0.20
Sarc (Clethrionomys only)		**11.49**	2.75	1.91	**4.26**	1.95	0.37
**B) Pathogen Richness** (Chi2, bold if significant)							
6 Pathogens	4.87	-	-	**4.33**	0.03	0.36	0.10
6 Pathogens + ectoparasites	**7.89**	-	**4.03**	**4.49**	0.86	0.13	0.10
**C) Pathogen community composition** ($R^{2,}$ bold if significant)							
All species	**0.12**	**0.07**	0.00	0.02	**0.03**	0.01	0.24
Apodemus only	0.02	0.01	0.02	**0.11**	0.06	0.02	0.24
Clethrionomys only		**0.20**	0.01	0.05	**0.07**	0.02	0.35

Table A7: Post-hoc pairwise comparisons between rodent species regarding flea occurrence, tick occurrence, richness of pathogens and ectoparasites and the pathogen community found in rodents' spleens. Shown are comparisons between group levels of the factor species with corrections for multiple testing. Significant differences are indicated in bold. For the pathogen community pairwise permutational multivariate ANOVAs were run as post hoc test, while all other post hoc comparisons are based on estimated marginal means (EMMs) to compare factor levels on the link scale (log-odds).

	Species pair	Estimate	SE	z.ratio	p.value
**Flea occurrence**					
	Cgla-Aagr	**2.19**	**0.81**	**2.70**	**0.019**
	Cgla-Afla	0.15	0.63	0.23	0.971
	Aagr-Afla	2.04	0.89	2.29	0.058
**Tick occurrence**					
	Cgla-Aagr	1.60	0.77	2.10	0.091
	Cgla-Afla	**3.75**	**1.14**	**3.28**	**0.003**
	Aagr-Afla	-2.15	1.20	-1.79	0.172
**Richness pathogens + ectoparasites**					
	Cgla-Aagr	0.32	0.17	1.84	0.157
	Cgla-Afla	**0.41**	**0.16**	**2.67**	**0.021**
	Aagr-Afla	-0.10	0.18	-0.56	0.842
**Pathogen community composition**				**F-value**	**p.value**
	Cgla-Aagr			**24.81**	**0.003**
	Cgla-Afla			**13.05**	**0.003**
	Aagr-Afla			1.23	0.274

Table A8: Complete test statistic for pathogen community analysis of pathogens found in the spleen in forest rodents, using three species instead of two genera. The outcome is very similar, compare main text, table 3B. Significant effects are highlighted in bold.

Factors	Df	SumOfSqs	R^2	F	Pr(>F)
Species	**2**	**1.76**	**0.12**	**5.20**	**0.002**
Season	**1**	**1.10**	**0.07**	**6.50**	**0.001**
Site	1	0.00	0.00	0.00	0.920
Sex	1	0.37	0.02	2.19	0.118
Emergence	1	0.09	0.01	0.56	0.630
Proportion sections	**1**	**0.51**	**0.03**	**3.00**	**0.043**
Residual	63	10.64	0.70		
Total	70	15.17	1		

Table A9: Details on rodent individuals that were included in the study.

ID	Year	Season	Habitat	Species	Sex	Weight
GEFWIN2110005	2021	autumn	forest	Cgla	f	13.60
GEFWIN2110006	2021	autumn	forest	Aagr	m	25.50
GEFWIN2110007	2021	autumn	forest	Cgla	f	20.80
GEFWIN2110008	2021	autumn	forest	Cgla	f	15.90
GEFWIN2110009	2021	autumn	forest	Cgla	f	18.50
GEFWIN2110010	2021	autumn	forest	Cgla	f	18.80
GEFWIN2110011	2021	autumn	forest	Cgla	m	19.10
GEFWIN2110012	2021	autumn	forest	Cgla	m	18.30
GEFWIN2110013	2021	autumn	forest	Cgla	f	21.70
GEFWIN2110015	2021	autumn	forest	Afla	m	44.90
GEFWIN2110016	2021	autumn	forest	Afla	m	37.90
GEFWIN2110018	2021	autumn	forest	Cgla	f	18.20
GEFWIN2110020	2021	autumn	forest	Cgla	f	21.10
GEFWIN2110023	2021	autumn	forest	Aagr	m	14.30
GEFWIN2110025	2021	autumn	forest	Afla	f	30.50
GEFWIN2110026	2021	autumn	forest	Afla	m	33.40
GEFWIN2110028	2021	autumn	forest	Afla	m	36.80
GEFWIN2110032	2021	autumn	forest	Cgla	f	20.65
GEFWIN2110037	2021	autumn	forest	Afla	f	29.70
GEFWIN2110046	2021	autumn	forest	Aagr	m	24.30
GEFWIN2110048	2021	autumn	forest	Cgla	f	24.00
GEFWIN2110049	2021	autumn	forest	Afla	m	40.10
GEFWIN2205002	2022	spring	forest	Afla	f	16.10
GEFWIN2205003	2022	spring	forest	Cgla	m	23.00
GEFWIN2205005	2022	spring	forest	Afla	m	17.00
GEFWIN2205006	2022	spring	forest	Afla	m	45.30
GEFWIN2205007	2022	spring	forest	Cgla	m	22.20
GEFWIN2205009	2022	spring	forest	Afla	m	15.90
GEFWIN2205010	2022	spring	forest	Cgla	m	27.30
GEFWIN2205012	2022	spring	forest	Afla	f	23.30
GEFWIN2205013	2022	spring	forest	Afla	m	42.00
GEFWIN2205014	2022	spring	forest	Afla	f	21.30
GEFWIN2205017	2022	spring	forest	Cgla	m	25.30
GEFWIN2205018	2022	spring	forest	Cgla	m	31.50
GEFWIN2205019	2022	spring	forest	Afla	f	14.70
GEFWIN2205021	2022	spring	forest	Aagr	m	32.60
GEFWIN2205022	2022	spring	forest	Cgla	m	11.60
GEFWIN2205023	2022	spring	forest	Cgla	f	27.70
GEFWIN2205024	2022	spring	forest	Cgla	m	25.20
GEFWIN2205025	2022	spring	forest	Cgla	f	15.00

GEFWIN2205026	2022	spring	forest	Afla	m	43.40
GEFWIN2205027	2022	spring	forest	Afla	m	37.60
GEFWIN2205028	2022	spring	forest	Cgla	m	33.10
GEFWIN2205029	2022	spring	forest	Cgla	m	31.50
GEFWIN2205030	2022	spring	forest	Aagr	m	33.00
GEFWIN2205031	2022	spring	forest	Afla	m	27.00
GEFWIN2205032	2022	spring	forest	Cgla	m	20.50
GEFWIN2205033	2022	spring	forest	Cgla	f	17.30
GEFWIN2205034	2022	spring	forest	Cgla	m	24.20
GEFWIN2205035	2022	spring	forest	Afla	m	43.10
GEFWIN2205037	2022	spring	forest	Cgla	m	18.90
GEFWIN2205038	2022	spring	forest	Cgla	m	28.10
GEFWIN2205039	2022	spring	forest	Aagr	f	7.80
GEFWIN2205040	2022	spring	forest	Cgla	f	17.50
GEFWIN2205041	2022	spring	forest	Afla	f	21.00
GEPBGP2110001	2021	autumn	park	Cgla	f	13.10
GEPBGP2110002	2021	autumn	park	Cgla	m	13.90
GEPBGP2110011	2021	autumn	park	Aagr	m	29.40
GEPBGP2110014	2021	autumn	park	Cgla	f	13.40
GEPBGP2110018	2021	autumn	park	Aagr	m	29.20
GEPBGP2205001	2022	spring	park	Afla	m	36.90
GEPBGP2205002	2022	spring	park	Afla	f	15.40
GEPBGP2205003	2022	spring	park	Cgla	m	17.00
GEPBGP2205004	2022	spring	park	Afla	m	36.00
GEPBGP2205005	2022	spring	park	Cgla	f	20.60
GEPBGP2205006	2022	spring	park	Afla	m	31.80
GEPBGP2205007	2022	spring	park	Cgla	f	9.70
GEPBGP2205008	2022	spring	park	Cgla	m	23.80
GEPBGP2205009	2022	spring	park	Cgla	f	13.10
GEPBGP2205010	2022	spring	park	Aagr	m	9.60
GEPBGP2205011	2022	spring	park	Aagr	m	NA
GEPBGP2205012	2022	spring	park	Aagr	m	NA
GEPBGP2205013	2022	spring	park	Aagr	m	NA
GEPBGP2205014	2022	spring	park	Cgla	m	NA
GEPBGP2205015	2022	spring	park	Aagr	f	NA
GEPBGP2205016	2022	spring	park	Afla	m	NA
GEPBGP2205017	2022	spring	park	Aagr	m	16.80
GEPBGP2205018	2022	spring	park	Aagr	m	31.50
GEPBGP2205019	2022	spring	park	Aagr	m	30.00
GEPBGP2205020	2022	spring	park	Cgla	m	17.70
GEPBGP2205021	2022	spring	park	Cgla	m	17.80
GEPBGP2205022	2022	spring	park	Cgla	m	14.90
GEPBGP2205023	2022	spring	park	Cgla	f	15.10

GEPBGP2205024	2022	spring	park	Cgla	m	13.30
GEPBGP2205025	2022	spring	park	Afla	m	43.40
GEPBGP2205026	2022	spring	park	Cgla	m	28.30
GEPBGP2205027	2022	spring	park	Cgla	m	23.50
GEPBGP2205028	2022	spring	park	Aagr	f	28.80
GEPBGP2205029	2022	spring	park	Cgla	m	17.30
GEPBGP2205030	2022	spring	park	Aagr	m	31.70
GEPBGP2205031	2022	spring	park	Aagr	m	32.60
GEPBGP2205032	2022	spring	park	Aagr	m	34.80
GEPBGP2205033	2022	spring	park	Aagr	m	33.90

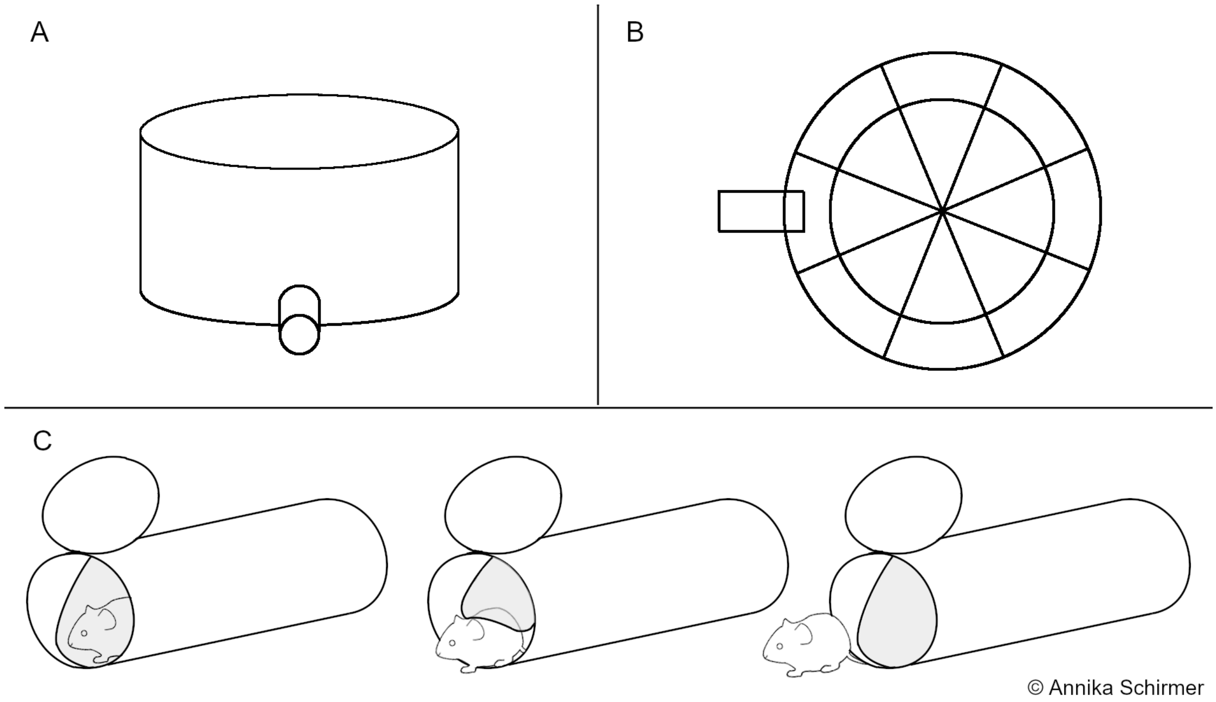

Figure A1 Overview of the behavioural test setup. A) Side view of the arena with the attached opaque pipe. B) Top view of the arena divided into 16 sections. C) Schematic representation of the door mechanism built into the tube end leading into the arena. The tube end leading into the arena was equipped with one opaque door (white), which was opened once the test began and a transparent, one-way inner door (grey) which could be opened by the tested individual itself. Once the individual passed the door, it closed automatically on its own preventing re-entering of the tube.
